# Identifying and Managing Suicidality in Myalgic Encephalomyelitis/Chronic Fatigue Syndrome

**DOI:** 10.3390/healthcare9060629

**Published:** 2021-05-25

**Authors:** Lily Chu, Meghan Elliott, Eleanor Stein, Leonard A. Jason

**Affiliations:** 1 Independent Consultant, Burlingame, CA 94010, USA; 2 Center for Community Research, DePaul University, Chicago, IL 60614, USA; meghan.elliott@depaul.edu (M.E.); ljason@depaul.edu (L.A.J.); 3 Department of Psychiatry, Faculty of Medicine, University of Calgary, Calgary, AB T2T 4L8, Canada; espc@eleanorsteinmd.ca

**Keywords:** severely ill, suicide screening, suicide assessment, suicide management, chronic illness, primary care, outpatient, adult

## Abstract

Adult patients affected by myalgic encephalomyelitis/chronic fatigue syndrome (ME/CFS) are at an increased risk of death by suicide. Based on the scientific literature and our clinical/research experiences, we identify risk and protective factors and provide a guide to assessing and managing suicidality in an outpatient medical setting. A clinical case is used to illustrate how information from this article can be applied. Characteristics of ME/CFS that make addressing suicidality challenging include absence of any disease-modifying treatments, severe functional limitations, and symptoms which limit therapies. Decades-long misattribution of ME/CFS to physical deconditioning or psychiatric disorders have resulted in undereducated healthcare professionals, public stigma, and unsupportive social interactions. Consequently, some patients may be reluctant to engage with mental health care. Outpatient medical professionals play a vital role in mitigating these effects. By combining evidence-based interventions aimed at all suicidal patients with those adapted to individual patients’ circumstances, suffering and suicidality can be alleviated in ME/CFS. Increased access to newer virtual or asynchronous modalities of psychiatric/psychological care, especially for severely ill patients, may be a silver lining of the COVID-19 pandemic.

## 1. Introduction

Myalgic encephalomyelitis/chronic fatigue syndrome (ME/CFS) is a debilitating chronic illness characterized by post-exertional malaise (PEM), unrelenting fatigue, unrefreshing sleep, cognitive dysfunction, and orthostatic intolerance. This illness is estimated to affect at least 0.42% of the United States (US) adult population [1]. ME/CFS causes significant reduction in functioning, and, as with many chronic illnesses, is associated with high rates of disability and unemployment [2,3]. Up to 69% [4] are unable to work and a quarter of patients report being consistently home- or bed-bound. Unfortunately, we do not yet understand the cause(s) of ME/CFS and there are currently no effective disease-modifying treatments: management is targeted at alleviating symptoms.

Multiple studies have found that people with ME/CFS are at an increased risk of death by suicide. In the UK, people with ME/CFS had a more than a six-fold increase in suicide risk (standardized mortality ratio 6.85) compared to the general population [5]. In Spain, 12.75% of people with ME/CFS, compared to 2.3% of the general population, were at risk of suicide [6]. Another study, despite not finding an increased suicide risk, discovered an increased risk of non-fatal self-harm [7], which is a robust predictor of future suicide attempt [8]. A retrospective convenience sample implicated suicide as one of the three leading causes of death in people with ME/CFS, alongside heart failure and cancer [9]. Compared to the general population, the median age at completed suicide was also significantly lower, at 39.3 years of age compared to 48 years [9]. Surprisingly, in a study focusing on the association between suicide and physical illness of all types in the United Kingdom, 16% of the deceased in the county sampled suffered from ME/CFS [10]. Among both moderately and severely ill ME/CFS patients, 39–57.25% [6,9,10] have contemplated suicide, compared to 4% of the general US population [11] and 1–10% of primary care outpatients [12]. 

Despite this situation being a clear, urgent public health issue, the specific reasons behind the increased risk of suicide in ME/CFS have not been well-examined. Emerging trends in the literature reflect the impact of not only the symptomatology of ME/CFS itself, but also the stigmatization of this illness by social connections (i.e., family members, friends, employers, etc.) [13], medical professionals [14] and the general public. Thus, those with ME/CFS may be at an even higher risk of suicide and mental health comorbidity than those with other chronic physical ailments, due to the additional burden of constantly having to justify, explain, and defend their disease experience [6]. 

Medical professionals who do not specialize in psychology or psychiatry nor work in a mental health setting play a vital role in suicide prevention and management. It has long been known that multiple or serious medical conditions subject patients to a higher risk of suicide. In the month preceding their completed suicides, approximately half of patients [15] saw a primary care provider at least once. In contrast, 71% had not had any contact with mental health services in the preceding year [16]. Tragically, even though many of these patients expressed suicide ideation or exhibited concerning behaviors during their last medical visit, most medical professionals upon later interview admitted dismissing or downplaying patients’ reports [15]. Medical professionals can not only identify people at risk, they can also prevent imminent suicides by directing high-risk patients to immediate/emergency mental health care. For patients determined to be at low- to moderate risk, they could potentially treat them within their medical clinics while collaborating with outpatient mental health providers and other specialists (e.g., medical social workers, occupational therapists, chronic pain management experts). 

**Figure 1:**
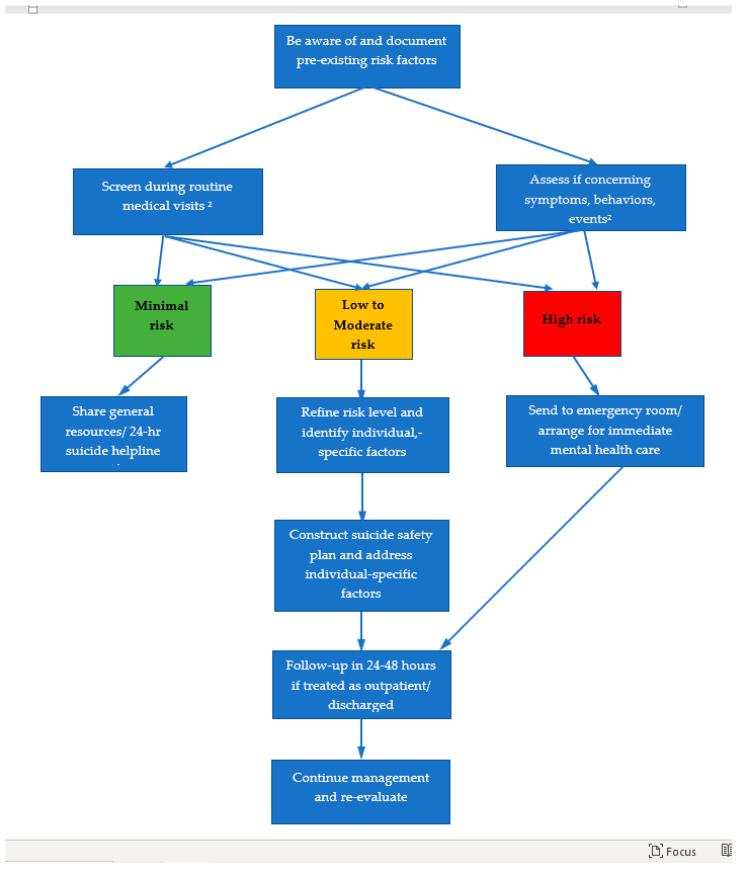
Overall approach to evaluation and management of suicidality in individuals. Use the Ask Suicide-Screening Questionnaire (ASQ) or Columbia—Suicide Severity Risk Scale (C-SSRS) for screening and assessment. See instruments for definitions of initial risk level and text for details.

The purpose of this article is four-fold: (a) to perform a review of the literature on ME/CFS and suicide, (b) to identify risk and mitigating factors for suicide in ME/CFS versus other chronic physical conditions, (c) to outline a strategy for assessing suicidality and (d) to explain basic management of at-risk patients in an outpatient medical setting. Because most ME/CFS research has involved adults, this article focuses on those 18 years of age and older. When we refer to clinicians in this article, we do not mean those professionals who specialize in mental health or work in such a setting (e.g., an internist assisting with medical issues in an inpatient psychiatric unit) but instead those who manage mostly physical health conditions. Figure 1 summarizes the process to assess and manage suicidality described in this article. 

## 2. Risk Factors for Suicide

To illustrate how information from this article can be applied, we have created a clinical case (Box 1), based on a composite of patients seen by one author (ES).

Box 1 **Clinical case—part 1.**

Maria is a 56-year-old woman who was diagnosed with ME/CFS 10 years ago. She experiences severe fatigue, nausea and dizziness upon sitting or standing up, problems with concentration and memory, and is easily overstimulated. Despite feeling exhausted, Maria describes her sleep as broken and unrefreshing. Her symptoms limit her activities to a total of 2–3 h daily out of bed. If she does more than 2 light household tasks (e.g., washing dishes, shopping for groceries), her symptoms worsen.

She has given up driving after nearly being in a serious accident due to slowed thinking and reaction time. She relies on the bus for medical appointments but the exertion of getting out of bed, dressing, waiting for the bus and being upright for the appointment leaves her exhausted and bed-bound for days. For the last 7 years, Maria has been unable to work as a manager at a telecommunications company.

Maria is divorced, lives alone and has no children. She has lost many friends because she often lacks the energy to get together even by phone. There is no one she can call on for practical help with groceries or rides. She has not been out for a social occasion for a few years. She receives a disability pension but it does not cover her monthly bills. She has drained her savings to supplement her pension.

### 2.1. What Factors in Maria’s Background Place Her at Higher Risk of Suicidality (e.g., Ideation, Attempts, Completed Suicide) Than the General Population?

Maria’s situation demonstrates a variety of factors which place her at higher risk of suicidal ideation, attempt, and completion. These factors can be classified by their modifiability and specificity to ME/CFS patients (vs. factors that are common in the general population or any chronically ill patient group). See Table 1 for a list of risk factors and their classification. Factors marked with an asterisk are especially relevant to ME/CFS patients, based on one study in Spain [6] and another in the United States [17]. 

Demographic and historical characteristics [12,18] are often chronic and not modifiable. History of a prior suicide attempt places patients at the high risk of a future attempt, even up to 3 decades later [19]. Compared to the general population, older and female patients are at higher risk of suicidal ideation although women are less likely than men to complete suicide. In the US and Canada, rates of suicide among people identifying as Native American/Alaskan or First Nations and lesbian, gay, bisexual, trans, or queer/questioning (LGBTQ) are respectively, 1.5–3 times higher than other ethnic/minority groups and 2–6 times higher than heterosexual peers [20,21,22]. Maria’s marital status, solo living situation, poor financial state, and lack of consistent social contact (whether via family, friends, or work) also place her at higher risk. In the United States, among non-depressed patients with ME/CFS, lack of resources, including social/financial support and occupational engagement, was the most cited reason (by 79%) contributing to suicidal ideation [17]. Among Spanish patients, lack of resources was linked to suicidal ideation, depression, and hopelessness [6]. Some of these adverse social factors can be ameliorated but will involve actions, professionals, and agencies beyond those strictly focused on healthcare. 

Conversely, medically related factors may be directly modifiable by the clinician even within just their own practice. In Maria’s case, persistent symptoms such as disturbed sleep, intense pain, severe limitation in function, and resultant poor quality of life can be addressed and managed further. These are factors that have been found to increase risk of suicidality across a variety of physical health conditions.

One hallmark symptom of ME/CFS is unrefreshing sleep [4], which is also a risk factor for suicide. Ahmedani et al. found that sleep disorders more than doubled the likelihood a person would die by suicide, compared to the general population [16]. In postural orthopedic tachycardia (POTS), often considered a sister disorder of ME/CFS, low sleep quality scores were significantly associated with suicidal ideation [23]. Although this is a troubling implication for suicide in ME/CFS, it also represents a salient opportunity for intervention; treating sleep dysfunction could be a practical way to reduce suicide risk and increase quality of life for people with ME/CFS. In a study of nonmalignant chronic pain—another common experience for those with ME/CFS—a significant indirect effect of chronic pain on suicide risk was found, mediated by disturbed sleep; with sleep removed from the model, the direct effect of chronic pain on suicide risk was nonsignificant [24].

Another highly relevant risk factor is functional limitation. Many chronic illnesses lead to reduction in daily activities; ME/CFS stipulates such a reduction as a criterion for diagnosis [4]. In a recent census-based study of people with chronic physical illnesses in Northern Ireland, the degree of functional limitation in chronic illness was the largest statistical predictor of death by suicide [25]. Those who reported that their day-to-day activities were “limited a lot” were more than three times as likely to die by suicide as those without functional limitations. In a qualitative study of people living with both multiple sclerosis (MS) and late-stage kidney disease, limitation of activities was mentioned by multiple participants as a driving factor in their suicidal ideation [26]. This finding has particularly salient implications for ME/CFS, which has been suggested in multiple studies to reduce function even more than MS [27,28]. Thus, one might expect to see an even greater risk of suicide in ME/CFS than in other chronic illnesses with less impaired functionality. Even within the ME/CFS category, there is a spectrum of physical functioning, with some individuals able to leave the house for work and recreation, while others with greater activity limitations are confined to their homes or even their beds.

The admittedly limited literature on ME/CFS and suicide seems to support functional limitation as a risk factor. Johnson et al. found that people with ME/CFS who were housebound were three times as likely to die by suicide than those who were not housebound, or, interestingly, those who were bedridden [29]. This finding suggests a floor effect, in which reduced functioning below a certain degree acts as a protective factor rather than a risk, possibly through limiting access to lethal means or due to the often-ubiquitous presence of a caregiver who, if understanding and non-stigmatizing, could provide additional social support as well as supervision. Such a floor effect could shed new light on mixed results from earlier studies of suicide in ME/CFS; for example, McManimen et al. did not find an increased risk of death by suicide for those with ME/CFS, but over half of the sample were bed-bound, perhaps explaining the lack of statistical significance in suicide risk [30]. Qualitative work in people who have ME/CFS but do not meet criteria for depression further underscores the role of functional limitation in suicide risk. Devendorf et al. qualitatively analyzed the open-ended responses of people with ME/CFS who endorsed suicidal ideation but did not meet criteria for depression. Reduced ability to participate in daily life was a common theme, with one person remarking [17]: “When crashed, I can do nothing but lie in my bed in total agony and in silence and darkness, trying not to move—sometimes for weeks on end. So, yes, that can be distressing and depressing and make it hard to concentrate or feel hopeful. But my desire for life and to participate in life has not changed. It’s not that I don’t want to do things; it’s that I can’t.”

Multiple studies suggest that presence of chronic pain, common in those with ME/CFS, is a significant risk factor for suicide. Braden and Sullivan found that the presence of any chronic pain condition was associated with both suicidal ideation and likelihood of suicide attempt. This association held true for both 12-month suicide risk (OR = 1.5, 95% CI 1.1–2.0) and for lifetime suicide risk (OR = 1.3, 95% CI 1.2–1.4) [31]. Interestingly, the type of pain which was associated most strongly with lifetime ideation, plan, and attempt, was the heterogeneous “other” chronic pain category, one which might be particularly relevant to ME/CFS due to its own heterogeneous nature. Chronic pain also feeds into the activity limitation suicide risk discussed previously; the combination of pain and fatigue could limit daily life even further. Fuller-Thompson and Nimigon, in a study of depression risk in those with ME/CFS, found that those whose activities were limited by pain were approximately 1.5 times as likely (OR = 1.59, 95% CI 1.11, 2.26) to have depression as those who did not experience such pain-related limitations [32]. Although pain treatment is a potentially promising target for intervention, it can become complicated when dealing with suicide risk; many commonly used pain medications are fatal in overdose, which must be considered from a reduction of lethal means perspective when addressing chronic pain and suicide.

### 2.2. What Risk Factors Are Unique or More Prominent in Patients with ME/CFS Compared to Patients Affected by Other Conditions?

There are also factors which are unique to ME/CFS. The unusual symptom of post-exertional malaise (PEM) can lead to and promote other risk factors. PEM refers to the appearance of new or worsening of baseline symptoms when patients engage in ordinary physical or cognitive activities, such as sitting upright, reading a newspaper article, or walking around the house [4]. PEM can occur immediately or be delayed by hours to days and can last hours to days, decreasing a patient’s function further. Thus, actions such as taking a walk outside to alleviate depression or meeting with friends to curtail isolation may not be possible. Cognitive dysfunction can manifest as a short attention span, poor memory, decreased comprehension, and word-finding difficulties, resulting in problems communicating and interacting with others. Although cognitive issues are one of the core criteria for ME/CFS, 31% to 45% of those severely affected reported this symptom as not only present but occurring at an intense level [33]. Hypersensitivity to stimuli—whether light, touch, sound, or substances (e.g., certain foods, fragrances)—has also been observed to be more common and intense in the severely ill compared to mildly and moderately affected patients [34]. In some patients, hypersensitivities play an equal or greater role than fatigue or post-exertional malaise in confining them to their homes. All these symptoms contribute to social and physical isolation and further limit function.

Moreover, since there are currently no disease-modifying treatments for ME/CFS, the root of Maria’s situation cannot be addressed directly yet. Instead, treatment is concentrated on managing ME/CFS symptoms and supportive care [35]. That can lead to feelings of frustration, disappointment, and hopelessness. Hopelessness is recognized as a risk factor for suicide in chronically ill patients and was cited by 48% of non-depressed people with ME/CFS contemplating suicide [17].

Additionally, since many healthcare professionals are not knowledgeable or continue to hold misconceptions about ME/CFS, patients often feel their experiences are dismissed, downplayed, or disparaged. For decades and up until a few years ago, ME/CFS was attributed to deconditioning [36] or to an irrational fear/avoidance of activity [37]. Homebound patients were characterized as “pervasively passive” “with a predominant belief in a somatic cause” while caregivers were blamed for “unwittingly contribut[ing] to the persistence of the condition by taking over too many activities of the patient.” [38]. Thus, patients were instructed that graded exercise therapy or ignoring/de-emphasizing their own symptoms via cognitive behavioral therapy (CBT) would lead to a cure or improvement. Some researchers and groups even discouraged or warned patients about joining ME/CFS support groups because the latter opposed these treatments [39,40].

We now know those theories are erroneous and even harmful: metabolic, neurologic, and immunologic abnormalities may underlie ME/CFS [4,41,42,43] and between 54–74% of patients have reported that their health worsened with exercise programs [44]. Nevertheless, these ideas and treatments persist as changes in the practice of medicine frequently take years to reach frontline practitioners. Lack of understanding from healthcare providers, being labelled as “rebellious”/“noncompliant” because they disagreed with now-disproven treatments, being blamed for their own illness, and the burden of having to educate others led to suicidal feelings, depression, and hopelessness among both US and Spanish patients [6,17]. In contrast, medical conditions such as multiple sclerosis, chronic heart disease, and stroke are recognized by the great majority of health professionals as legitimate, severely disabling diseases. Patients can rely on their professionals’ knowledge, experience, and sympathy. Many communities even have specialty clinics and designated support services available for these conditions.

Lack of knowledge and negative attitudes also permeate the public’s view of ME/CFS. For those living with this illness, such ignorance and stigma can lead to a variety of distressing encounters, even with those considered close social contacts. McManimen et al. found that people with ME/CFS who met depression criteria or endorsed suicidal ideation were more likely than those who did not meet criteria to have experienced unsupportive social interactions—both overall and on specific distancing, minimizing, and blaming subscales—and stigma [13]. This finding suggests that the dismissive, harmful interactions experienced by those with ME/CFS might contribute to the increased suicide risk. In a study comparing people with ME/CFS and/or fibromyalgia to those with an autoimmune disorder, the overall level of unsupportive interactions did not differ, but the nature of such interactions did; those with ME/CFS were significantly more likely to report “distancing” and “minimizing” interactions [45]. People with ME/CFS were, in one study, more likely to report never having been married than those with other chronic illnesses [46], suggesting an illness-specific hindrance of social relationships. In a study of a suicide risk scale for people with POTS, a disorder related to the orthostatic intolerance symptom of ME/CFS, 79% of respondents reported “high” or “very high” loneliness on the UCLA Loneliness Scale [47], further suggesting social impoverishment in ME/CFS as a mechanism for suicide risk. The trend of lack of social support suggests that education and stigma reduction could be a powerful mechanism for suicide risk reduction at both the individual and global levels.

## 3. Initial Screening/Assessment of Suicide Risk: Is This Patient Currently at Risk of Suicide?

### 3.1. Who Should Be Assessed for Suicidality and When Should It Be Done?

Some organizations, such as the Joint Commission recommend screening all adult medical patients for suicide [48] while others such as the United States Preventive Services Task Force (USPSTF) and Canadian Centre for Addiction and Mental Health found insufficient evidence for universal action [49,50]. Universal screening may not identify more at-risk patients than selective screening and it is unclear whether earlier intervention is effective. Selective screening targets patients with one or more risk factors. As illustrated by our clinical case (Box 1), patients affected by ME/CFS would certainly fit into this category. Before screening is instituted, clinicians should prepare a reference sheet of local mental health professionals, institutions, and resources they can refer to quickly should patients screen positive and be at a high risk of suicide [51]. Some patients may be reluctant or, if extremely ill, unable to describe their circumstances: reports from family, friends and caregivers should be heeded.

Ideally, all patients with ME/CFS should be screened upon initial intake and then occasionally through the years, perhaps linked to when other preventive measures are being discussed or carried out (e.g., annual influenza vaccination, mammograms, etc.). Conducting screenings during these times can be framed as part of the process the clinician and/or clinic regularly performs for all patients. Acceptability of screening is high with between 81–95% of patients [52,53] deeming it to be an appropriate component of inpatient and outpatient medical care.

Although some patients will directly report suicidal thoughts or behaviors during an office visit, up to 81% of people who saw their physician shortly before dying did not. However, reviews of the medical records and interviews with clinicians suggest premonitory signs [12]. Certain beliefs, statements, symptoms, and actions expressed by patients should prompt more immediate screening (Table 2). Feelings of hopelessness, loneliness, disconnectedness from others, and being a burden to society [54] have been linked to increased suicide risk among the chronically ill. Statements directly or indirectly surrounding these feelings should be explored further. Mood changes encompass new onset of depression and anxiety, exacerbation of pre-existing mood disorders, and rapid/intense fluctuations. People who exhibit agitated or impulsive behaviors may be more likely to attempt suicide rather than merely confining themselves to thoughts [55]. Clinicians should also pay attention to worsening or relentless chronic pain, sleep, or other symptoms. Patients who suddenly seem more peaceful without a clear cause after a period of depression should be assessed especially carefully: their lightening of mood may be due to finally deciding to proceed with a suicidal plan.

Another trigger to query patients is when major negative events happen, singularly or in quick succession. Examples of such events are divorce, unemployment due to disability, sudden worsening of health, denial or loss of disability benefits, failure of a highly anticipated treatment, and threat of homelessness. Other times, without an inciting event, patients may simply become weary of their unrelenting symptoms and difficult circumstances. Indeed, although they may occur for reasons unrelated to suicide, abrupt cessation of treatments, withdrawal from care and avoidance of contact with health professionals have been flagged as potential warning signs [55]. If a previously engaged patient suddenly disappears, health professionals should explicitly ask why: is it due to a mood disorder and/or hopelessness or for more mundane reasons (e.g., cost of care, preference for another provider)? The period immediately after a recent suicide attempt or discharge from inpatient/outpatient psychiatric/psychological care is also acknowledged to be perilous times.

A third pattern is acute-on-chronic distress. A patient may be coping to some degree with chronic risk factors but then be blind-sided by additional events (Box 2).

Box 2 **Clinical case—part 2.**

Recently, Maria underwent cervical spinal stabilization surgery for left arm pain, numbness, and weakness. Although she regained use of her arm, she is left with chronic pain which gradually spreads from her left arm to her whole body. Her building had a water leak and her apartment suffered water damage. Maria developed a rash, difficulty breathing and increased general unwellness. She suspects mold growth but was unable to afford to have it investigated. The building management has denied there is a problem. Maria wants to move but her limited health, financial means and social connections make it hard to do so.

Maria reports feeling hopeless at her appointment today. She says she does not want to endure additional health problems. It all feels too much to handle. She cannot think of anything she enjoys any more.

In Maria’s case, she appeared to be coping somewhat until development of new symptoms (widespread pain, breathlessness, rash) and disruptions to her housing situation. Through careful listening and observation of a patient, health professionals can initiate a conversation by calmly stating “The last few weeks sound really challenging. How have you been coping?” and then seeing how the patient responds. Some, such as Maria, will openly and willingly share how they feel (e.g., hopeless, weary, and anhedonic) in which case the health professional should let them talk uninterrupted.

It bears repeating that asking or talking about suicide with patients does not stimulate new or encourage existing thoughts of suicide. Both a 2014 review [56] and a 2018 meta-analysis [57] addressing this issue found no significant risks from screening; suicide risk and attempts were slightly reduced instead. Many people will feel relieved that the practitioner is taking their distress seriously. Such discussions are also not futile. People who attempt or survive suicide often demonstrate ambivalence about their actions [58]. Some part of them wants to live: most dramatically, all 29 people who survived a jump from the Golden Gate Bridge recounted they regretted their decision as soon as they stepped off the bridge [59,60]. For chronically ill people, suicide may be less about ending their lives than ceasing their symptoms and the downstream consequences. For example, Anne Ortegen, afflicted with severe ME/CFS, noted she still retained her joy and curiosity about life but “unbearable [physical] suffering” with no effective treatment in sight compelled her to seek medical aid in dying [61].

Unfortunately, some physicians and authorities, erroneously attributing ME/CFS symptoms to psychiatric/psychological etiologies, have forcibly detained patients in inpatient psychiatric units. Children [62] and severely affected adults have been especially vulnerable since they are unable to advocate vigorously for themselves. For example, Sophie Mirza, whose health was declining rapidly, was labelled as suicidal and subsequently hospitalized. It took her family much time, expense, and legal maneuvering to obtain her release. When she passed away, an autopsy discovered significant inflammation of her spinal cord, which might have contributed to her condition [63]. Thus, patients may be rightfully reluctant or fearful of admitting to suicidal thoughts or actions. Clinicians can reassure patients by framing assessments within the context of chronic medical illness (e.g., “Many chronically ill patients, like my patients with heart or lung disease occasionally experience suicidal feelings. Have you felt similarly?”) and informing them that suicidality can often be cooperatively managed with the patient maintaining agency in an outpatient setting. Contemporary mental health standards encourage care in the least restrictive manner and setting possible [64].

### 3.2. How Should Patients Be Screened or Assessed? What Issues Should Clinicians Pay Attention to or Ask about?

Since many medical professionals do not feel confident assessing suicidality [65,66], we recommend using validated, standardized instruments as much as possible. For routine screening, self-administered versions of these instruments can be used, or the questions asked by ancillary staff (e.g., medical assistants) but for more urgent situations, the healthcare professional should ask the questions themselves. Severely affected patients may need help from their caregivers with completing the questionnaires or their caregivers may have to serve as proxy respondents. Questionnaires may need to be administered over the telephone or via virtual visits. Using these tools assures that salient issues are covered using validated questions, patient responses are appropriately interpreted via an expert-vetted scale, and thorough documentation of this crucial conversation exists.

**Figure 2:**
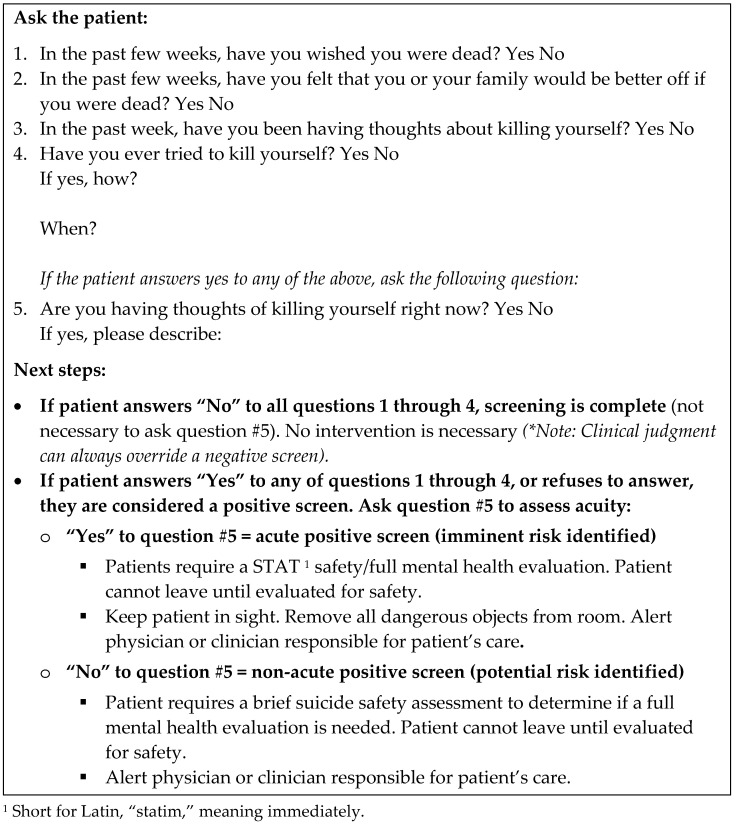
Ask Suicide-Screening Questions (ASQ). Reproduced with permission from Dr Lisa M. Horowitz, Arch Ped Adolesc Med, published by JAMA Network, 2012 [67].

**Figure 3:**
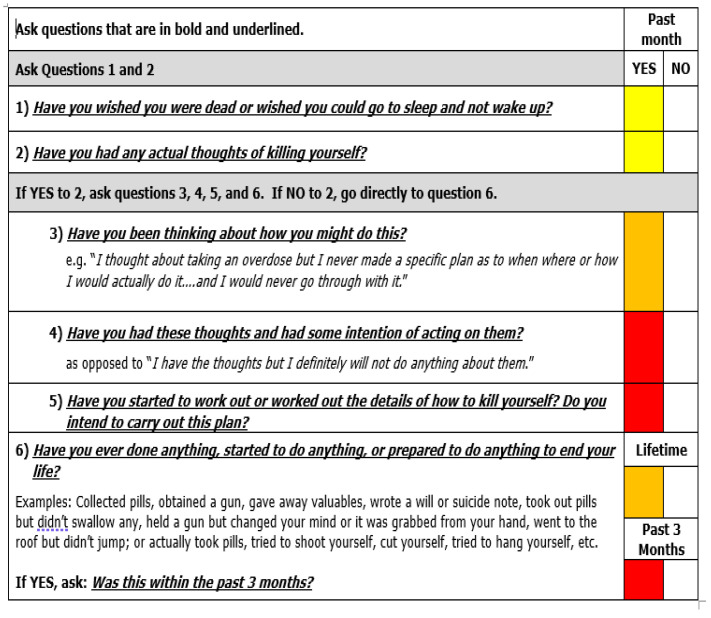
Columbia-Suicide Severity Rating Scale (C-SSRS): screen with triage points for primary care. Pay attention to question 2: if “Yes”, ask questions 3 through 6; if “No”, ask question 6. “Yes” responses in white boxes signify minimal risk; yellow boxes, low risk; orange, moderate risk; and red, high risk. Patients should be classified according to the highest risk box to which they reply “Yes”. Reproduced with permission from Dr Kelly Posner Gerstenhaber. The Columbia Lighthouse Project (https://cssrs.columbia.edu/ (accessed on 17 May 2021)).

Currently, there is a lack of brief, validated instruments for screening or assessment specifically created to be used by medical professionals in busy clinic settings. Two recent candidates are the US National Institute of Mental Health’s Ask Suicide-Screening Questions (ASQ) [67,68] (Figure 2) and Columbia-Suicide Severity Rating Scale (C-SSRS) [69] (Figure 3). Both instruments take only minutes to administer (5 and 6 questions respectively), address passive and active suicidal ideation (both types confer equal risk), ask simple Yes/No questions initially, and prompt patients for more details. If patients are unable or unwilling to answer any question on the ASQ, the default is to score the question as though the patient answered “Yes”. Although 4 of the 5 ASQ’s questions concern thoughts, the C-SSR covers thoughts, methods, intentions, and specific plans. “Yes” responses on each subsequent question are linked to escalating suicide risk. Stronger intentions and more recent, explicit plans indicate a higher risk [18,70]. Note both instruments inquire about lifetime history of suicidal actions [71]; unlike suicidal ideation in the remote past, actions anytime in the past significantly increase risk of another attempt. 

Both instruments superbly separate out non-suicidal patients; however, the ability to identify at-risk patients accurately is mixed. Negative predictive values are the percentages generated when patients whose questionnaires indicate minimal risk and who truly are minimal risk (true-negatives) are divided by the total number of patients (both true- and false-negatives) determined by the questionnaire to be at minimal risk. Positive predictive values are calculated the same way except they concern patients whose questionnaires indicate some risk of suicide. For the C-SSRS, among psychiatric or non-psychiatric subjects enrolled in a variety of clinical trials, negative predictive values (NPV) for prospective suicidal behavior ranged from 97.93% to 99.63%. In contrast, the positive predictive values (PPV) are quite low, ranging from 8.97% to 16.49%, with the higher values observed in psychiatric patients [72]. For the ASQ, in medical and surgical inpatients, compared to the longer, more detailed Adult Suicidal Ideation Questionnaire, the NPV was 100% and the PPV was 32% [68]. 

These low PPVs are in-line with other suicide assessment instruments [73] and may not be as alarming as they first seem. Predictive values are substantially influenced by the prevalence or absence of a condition in a population. As the prevalence of a condition increases, the PPV increases whereas the NPV decreases. Suicidal attempts and completions, not ideation, in primary care outpatients are still relatively rare, with a prevalence of 1% [18] or less. Thus, PPVs will be low. The prevalence is likely higher among chronically medically ill patients (e.g., 8.9%) [74]: with that increase, the PPV will increase. Secondly, predicting future suicide attempts might not be the best measure of these instruments’ effectiveness. Assessment and screening per se do not prevent or stop suicide attempts. Instead, these actions help clinicians to identify who needs further evaluation and to design individualized care plans. If the plan works as intended, suicide attempts may never be made. Conversely, if the patient is resolved to commit suicide, attempts and completed suicides will result. 

### 3.3. Why Should Clinicians Screen for Suicide Directly, Independent of Mood Disorders or Anxiety?

Confronting suicide directly (Box 3) and not only within the context of depression or anxiety is important. Although both the ASQ and C-SSRS’s questions appear blunt, they avoid confusion or equivocation. In the past, 90% of suicides were attributed to psychiatric disorders [75]. Consequently, suicide evaluations customarily take place during examination for mood disorders. One popular method uses item 9 of the Patient Health Questionnaire-9 (PHQ-9) [76] which pertains only to the frequency of suicidal thoughts. However, up to 30% of patients endorsing absence of suicidal thoughts may currently be at risk [77] while 16–19% endorsing any presence may not be [77]. In one study, the PHQ-9 flagged 24% of subjects as suicidal versus the 6% and 1% detected, respectively, by the C-SSRS and clinical interviews [77]. Secondly, as mentioned in the section on risk factors, chronically ill patients may be suicidal for reasons unrelated to a mood disorder. The 90% figure cited previously originated from controversial “psychological autopsies” [78] (e.g., interviews of surviving family members) after completed suicides and closer examination in one study found only 17% were linked with depression [79]. More recent data from the US Centers for Disease Control and Prevention indicate that 54% of those dying by suicide in the US do not have any known mental health condition [80]. Third, steps to prevent suicide can be implemented right away and go beyond treating psychiatric disorders, as discussed later in this article. Conversely, it may take weeks to months for patients to start seeing the benefits of some depression and anxiety treatments.

Box 3 **Clinical case—part 3.**

Maria’s doctor administers the C-SSRS. Maria admits in the last 2 weeks, she has wished she would develop a fatal illness to put an end to what she views as a senseless existence. She occasionally thinks about taking more of the pain pills she was prescribed but explains that she does not have the courage to commit suicide. She worries that if she messes up she could be left worse off. She denies having the means to kill herself. She does not own any firearms and has not stockpiled medications. Maria has no history of suicide attempts.

## 4. Secondary Assessment of Suicide

### 4.1. Is This Patient at Low, Moderate, or High Risk of Suicide?

A clear advantage of both questionnaires is they quickly help clinicians determine if and how urgently management is needed, along with the best setting/professionals for care. If patients answer “No” to the first 4 questions of the ASQ [68] (Figure 2), the clinician does not need to ask the remaining question 5. For the C-SSRS [69] (Figure 3), a color-coding system is used with white, yellow, orange, and red boxes checked “Yes” indicating, respectively, minimal, low, moderate, and high risk. If the patient denies C-SSRS question 2, question 6 should be asked next. Absence of preparatory suicidal behaviors in their lifetime and in the last three months classifies the patient as minimal risk (i.e., only white boxes are marked). Responses should be recorded but no specific, current intervention is needed for patients falling in this category.

Conversely if patients answer “Yes” to question 5 of the ASQ or any one of questions 4, 5, and 6 (within the last 3 months) of the C-SSRS, these patients are deemed high risk. Immediate care needs to be instituted, whether that be a referral to the emergency room or a same-day appointment with an outpatient mental healthcare professional for urgent, comprehensive care. High-risk patients should not be left alone at any time and should be watched by a family or staff member whether they are at home or in the clinic. The patient’s clothes, possessions, and environment should be searched and cleared of any potentially harmful/lethal implements (e.g., guns, pills, knives, ropes/rubber tubes). If transportation to the emergency room or other facility is needed, consider calling an ambulance rather than having the patient or family member drive themselves or take public transport. If the patient interview suggests they have acted, ask the patient directly if they have done anything to hurt themselves already, such as overdosing on sedatives or cutting themselves. These behaviors may require immediate medical and not just psychiatric care. 

Based on Maria’s answers on the C-SSRS, answering “Yes” to questions 1, 2, and 3 but “‘No” to questions 4, 5, and 6 (Figure 3), she would be classified as at moderate risk (Box 4). For the ASQ (Figure 2), this category would apply to those answering “Yes” to any of the first four questions but “No” to item 5. Patients answering only “Yes” to questions 1 and/or 2 on the C-SSRS are deemed low risk. These patients should undergo further appraisal by the clinician to refine the level of risk, decide upon urgency of outpatient mental health care referral and identify modifiable risk factors. Although they may not require emergency room to services or a mental health appointment that day, there are still actions the clinician should take during the visit. 

Regardless of how patients are classified during this initial stage, the clinicians should thank the patient for trusting them enough to share these intimate and often painful experiences. All patients should be provided the contact information for the national 24-h suicide prevention hotlines in their country. For the United States, this number is 800-273-8255 (https://suicidepreventionlifeline.org/ (accessed on 17 May 2021)); for Canada, 1-833-456-4566 (https://www.crisisservicescanada.ca/en/ (accessed on 17 May 2021)); and for other countries, see https://www.opencounseling.com/suicide-hotlines (accessed on 17 May 2021)).

Box 4 **Clinical case—part 4.**

Based on her C-SSRS score, Maria appears to be at moderate risk of suicide.

### 4.2. How Can Risk Be Further Evaluated, Especially for Patients Deemed to Be at Moderate Risk?

Review factors that increase (Table 1) and reduce risk (Table 3) with the patient if they have not been documented previously or have not come up during the current visit. The developers of both the ASQ [70] and C-SSRS [81] also provide similar lists on their websites. Because the ASQ concentrates on suicidal thoughts but not current intention, plans, or actions, the secondary level of risk assessment encourages clinicians to discuss these topics with the patient.

Taking a thorough social history including where the patient currently lives; who they live with; whether they are engaged in work, school, or other activities; who they can rely on for regular support, logistical and psychological; and non-medical stressors in their lives (e.g., employment, divorce, bankruptcy, homelessness) can be revealing. Because ME/CFS, by definition, substantially impairs daily function, ask particularly about any difficulties performing basic (e.g., ambulating, toileting, bathing, etc.) or instrumental (e.g., cooking, shopping, managing medications, etc.) activities of daily living [82]. Ask even if a patient appears functionally normal during an appointment. To interact with clinicians, patients often save up energy before a visit or, conversely, plan to suffer the consequences of any physical/cognitive exertion afterwards.

All patients should be examined for depression and anxiety using standardized, validated instruments such as the Patient Health Questionnaire-2 (PHQ-2), General Anxiety Disorder-7 (GAD-7), or Hospital Anxiety and Depression Scale (HADS) [83,84,85]. Instruments emphasizing fatigue, insomnia, decreased activity, or appetite—symptoms which may be due to ME/CFS itself—may mistakenly label patients with major depression. Furthermore, the most current definition of ME/CFS, created by the National Academy of Medicine in 2015 [4], includes the symptom of orthostatic intolerance (OI). Upon sitting or standing up for periods as short as a few minutes, patients may report dizziness, nausea, confusion, heart palpitations, and short of breath. These symptoms disappear or improve upon lying down but mimic those of anxiety. Hence, it is no surprise that patients affected by OI have been misdiagnosed with anxiety [86]. The HADS has been used in several ME/CFS studies and all three instruments focus on affective rather than somatic symptoms, thus reducing the risk of overdiagnosing psychiatric disorders.

If not expressed, assess for loneliness, thwarted belongingness, and burdensomeness. Items originating from a suicide-screening instrument by Pederson et al. [47] intended for patients specifically affected by “invisible” illnesses (i.e., those without immediate, visual clues of illness, such as weight loss or jaundiced skin) could be adapted. Clinicians can ask: “How often do you feel lonely?” “Do you feel like you are a burden to your family or caregivers?” “Do you have a sense of belonging within your family?“ “Is there anyone or any group you feel connected with?”

Conversely, review protective factors (Table 3) with the patient. If not voluntarily offered (e.g., “Even though I feel bad, I would never kill myself. My children need me.”), ask patients directly “What would keep you from harming yourself?” “What makes your life worth living?” Some factors such as being a parent or being married are outside of the clinician’s hands while others can be introduced or reinforced, such as positive coping behaviors.

There is no threshold upon which a patient’s risk of suicide can be confirmed, downgraded, or upgraded. Instead, clinicians will need to examine the quantity, nature, and intensity of risk and protective factors to decide a patient’s final status (Box 5). The patient’s final status and the rationale for it should be documented in the medical records. The clinician’s intuition about a patient/situation should always override any answers on questionnaires or checklists.

Box 5 **Clinical case—part 5.**

Maria is assessed for anxiety and depression-related disorders. She scores 18/27 on the PHQ-9, a score suggestive of moderately severe depression. Her scores on the HADS are 15 for depression and 11 for anxiety, both in the clinical range. She has never smoked, rarely drinks, and denies abuse of other substances. She denies impulsive behaviors.

Her other risk factors are her chronic medical problems (including ME/CFS, pain, unrefreshing sleep, possible OI, and breathing problems), hopelessness, functional limitations, social isolation, poverty, and unstable housing situation. Her protective factors include her religious faith, her belief that she is not brave enough to commit suicide and a clinician she feels comfortable speaking with. Overall, her risk level of moderate remains unchanged.

## 5. Managing Suicidality

### 5.1. What Steps Would You Take Next? What Are Interventions All Suicidal Patients Should Receive?

A 2-step approach incorporating both general management of suicidality as well as management of patient-specific factors can be implemented. The approach outlined here applies mostly to patients at low to moderate risk who are suitable for outpatient treatment. For high-risk patients, their initial care may be carried out by the emergency room and/or an inpatient psychiatric unit. After discharge, similar steps can be used for these high-risk patients if they have not already been initiated by prior clinicians.

General management consists of (a) referring patients to mental health professionals and (b) collaborating with patients to create a suicide safety plan. As mentioned earlier, clinicians should generate and maintain a list of local mental health professionals, facilities, and resources so that referrals can be made as quickly and seamlessly as possible. Ideally, patients should be seen or contacted within 48–72 h. Psychiatrists can help with pharmacologic management of depression, anxiety, and some symptoms such as sleep. However, even patients without anxiety or depression can benefit from mental health care [87]. Psychological treatments using dialectical behavioral and cognitive behavioral therapy processes as well as a new therapy called Collaborative Assessment and Management of Suicidality are designed to specifically address suicidality [88]. Clinical trials and observational studies show these treatments decrease suicidal thoughts and attempts by 37.5% to 60% [88,89].

Before patients leave their office or end the visit, clinicians should collaborate with them to create a suicide safety plan [90]. Like diabetes “sick day” or asthma action plans most medical providers are already familiar with, these are written documents the patient can easily look to for guidance when suicidal thoughts and feelings surface or intensify. These plans are not just practical by themselves; they also help the patient feel more in control by reminding them of the alternative actions they compiled [90,91]. “Tunnel vision” or psychological constriction, whereby patients feel trapped and cannot see other options, is well-recognized among suicidal patients [58]. Engaging in alternative actions also allows impulsive thoughts to dissipate [92].

In 2008, Brown and Stanley produced a 6-step suicide safety plan which clinicians and patients can readily complete together [92]. Other staff (e.g., nurses, medical assistants, etc.) can also be trained to help patients fill out the form. Table 4 lists these 6 components, sample questions to elicit responses, and examples of how patients might answer. For a paper template that can be immediately printed out and used, see or the suicidesafetyplan.com website. Encourage patients to be as detailed as possible and to fill in steps using their own words. However, if a patient does not find one of the steps useful for them, they can skip it. People named in “Step 3” do not necessarily need to know of the patients’ suicidality whereas those in “Step 4” and “Step 5” might already know or can be informed by the patient. Firearms are a common and lethal method of suicide in the United States, so Step 6 should always include questions about access to handguns and rifles. Since patients with ME/CFS often take sleep, pain, or other medications, ask about which drugs they have, whether they have stockpiled tablets and how they are handled. Additionally, customize the answer: if a patient brings up leaping from a bridge, make sure Step 6 addresses that method. Although not a step, ask patients to record their reasons for living on the form. Finally, ask patients how likely they are to carry out the steps and what obstacles they might encounter. If needed, revise the plan so it will be simple to actualize.

Completion of the form is estimated to take between 30 to 45 min. Afterwards, several copies should be made, including one for the clinician’s medical records, and several copies for the patient to be stored in convenient locations. For example, a miniature copy on their nightstand or a scanned version on their mobile phone might be easier to find than a paper form in a desk drawer. Obtain written permission to share the plan with the patient’s supporters so they can reinforce the steps. For more information about how to use the template, visit suicidesafetyplan.com. Some clinicians express not knowing how to ask about means and recommending ways to restrict them [93]: the Suicide Prevention Resource Center provides free online training via their Counseling on Access to Lethal Means program [94].

Compared to patients receiving usual care, patients introduced to suicide safety planning during an emergency room visit were half as likely to attempt suicide in the subsequent 6 months [95]. Furthermore, two thirds of this group cited the plans as instrumental in reducing their suicide risk and twice as many showed up for follow-up mental health appointments as those in the usual care group. In contrast, although they have been recommended for many years, there is no consistent evidence of effectiveness for no-suicide contracts, whereby the patient promises the clinician they will not take action [96]. Although such contracts may make clinicians feel more secure, they do not help patients in crisis and may even compel patients to conceal intense feelings, out of a misguided effort to avoid disappointing the clinician.

The 2-step approach is illustrated in Box 6.

Box 6 **Clinical case—part 6.**

Maria’s doctor informs her that based on what she has expressed, her medical/social situation, and the questionnaire results, she is at moderate risk of suicide. She introduces Maria to the purpose of suicide safety plans and together they start completing one. Initially, Maria cannot think of calming activities nor who she can count on for support. With a little more probing, she remembers she dropped her knitting hobby after work became too busy and that her neighbor Sarah has said to call any time to chat. While they are completing the form, the doctor asks the receptionist to set up an appointment in the next 2 days with a psychiatrist, Dr Joseph Lopez, who offers virtual appointments in his practice.

The medical assistant prints out materials about suicide with the national suicide prevention hotline and a local helpline on them. She gives Maria the pamphlet.

### 5.2. What Are Individual-Specific Suicide Risk Factors? How Should They Be Addressed?

Individual-specific factors refer to those characteristics covered in Table 1 and in the secondary suicide risk assessment evaluation. They are called “individual-specific” because not every suicidal patient is affected by them nor is their degree of influence the same for each patient. Some factors even increase risk in one patient but are protective in another. For example, a happy marriage is protective but one marred by conflict or abuse is not. A well-paying meaningful job might be protective while a low-paying, stressful position may be worse than unemployment. Thus, based on what the patient reports, the clinician will need to make a judgment about how to classify a factor. Table 5 shows one way to categorize patient-specific factors, examples of factors, and examples of interventions to address them.

Two factors that require immediate treatment regardless of the presence of others are anxiety and depression. As mentioned above, while depression does not explain all suicides, it may be linked to 17% to 54% of them [79,80]. Between 21–88% and 17–47% [97,98] of patients with ME/CFS may be afflicted by anxiety or mood disorders, respectively. HADS scores above 11 indicate presence of anxiety or depression: Maria’s results indicate she is affected by both. Pharmacologic treatment of depression and anxiety for people with ME/CFS is no different from those without it [97,99]. As with treatment of any patient with a chronic medical condition, clinicians should avoid medications that could interact with existing medication and exacerbate ME/CFS symptoms (e.g., cognition) while favoring medications that may serve dual purposes (e.g., citalopram can be used for both anxiety and depression). Some patients may react strongly to medications, especially the severely ill: thus, starting at a lower dosage than usual and titrating up slowly is wise.

Although psychological treatments such as CBT are not recommended for ME/CFS, they are moderately effective for mood disorders and anxiety disorders [100,101]. Maria should be referred to a counselor or therapist who can support her over the short to medium term. If possible, select mental health professionals who regularly treat patients with chronic, disabling medical illnesses and who are familiar with or willing to learn about ME/CFS. Patients may need to be convinced that psychological treatment is not being prescribed for ME/CFS itself. Tell them you can refer them to another mental health professional if the initial practitioner is not compatible with them.

Ideally, mental health professionals should be notified that common solutions for other patients such as physical exercise, increased socialization, intense “homework” (i.e., complicated, long workbooks), and relaxing music might not be possible or will need to be adapted to patients with ME/CFS due to symptoms such as PEM, cognitive dysfunction, and hypersensitivity to sound. Due to their decreased mobility, moderately and severely ill patients would benefit greatly from therapy delivered remotely via telephone and video-conferencing: fortunately, these modalities appear to be comparably effective [102] to sessions administered in person. Shorter but more frequent visits, asynchronous interaction, and/or written vs. oral may be beneficial. For an in-depth discussion of assessment and treatment of psychiatric issues in ME/CFS, see author ES’s articles [97,99].

As with any patient affected by multiple, chronic, and/or complex conditions, care will often take place over time and multiple visits. The clinician will need to prioritize which factors to address and which interventions to start right away versus which ones can wait. First, ask patients “What can we do or change that would make your life worth living?” The answers given might be surprising and engages the patient in planning care. Other criteria might be the urgency of a factor (e.g., impending homelessness), how quickly the intervention may start working (e.g., pain medication), and how common the factor has been found to influence suicide risk among people with ME/CFS (e.g., lacking healthcare providers knowledgeable about ME/CFS).

There are a few caveats when introducing remedies similar to those in Table 5. Patients affected by ME/CFS may be exquisitely sensitive to the active or inactive components (e.g., coloring agents, preservatives) in a medication. Thus, start low and titrate up any medication slowly. Physical activity performed to alleviate co-morbidities (e.g., fibromyalgia, postural orthostatic intolerance syndrome) or during assessments (e.g., physical therapy) should be adapted so patients avoid triggering PEM. For severely ill patients, physical activity may not be possible without making the patient sicker. Although pain (e.g., joint/muscle aches, sore throats, headaches) is common in ME/CFS [103] and sleep disturbances are part of diagnostic criteria for ME/CFS [4,104], clinicians should evaluate the cause of symptoms before attributing them solely or entirely to ME/CFS, especially if the pain is new or worsening. For example, fibromyalgia, migraine headaches, and obstructive sleep apnea are common comorbid conditions yet each condition has specific treatments. There have been rare cases where late-stage cancer was discovered to be the source of pain. If possible, attempt to refer patients to other professionals who are knowledgeable or open to learning about ME/CFS.

The significance of validation and understanding conveyed by even one supportive clinician cannot be emphasized enough. Most patients affected by ME/CFS have endured years of indifferent or degrading medical professionals [6]. Even if a clinician is not adept at caring for ME/CFS patients, any good-faith efforts to learn about ME/CFS and communicate sympathy will be appreciated. During a short appointment, clinicians can earn the trust of patients by carefully listening to them, reflecting back to them their understanding of what was said, and honestly admitting what they know or do not know. There are many steps (Box 7) clinicians can take to improve their relationship with patients [105]

Box 7 **Clinical case—part 7.**

The doctor considers starting Maria on an antidepressant but decides to let the psychiatrist start a medication. When asked which symptoms are the most problematic, Maria nominates pain and sleep. When asked what else would improve her quality of life immediately, Maria desires help with daily tasks like bathing and the ability to be a little more social.

A week’s supply of a low dose of amitriptyline, which addresses both sleep and chronic neuropathic pain, is called in by the medical assistant to a pharmacy that delivers. Maria is told the medication may take up to 4 weeks to start working. The doctor also tells Maria she will ask a physical therapist, occupational therapist, and medical social worker to visit her at home.

On her way out of the office, Maria is stopped by the receptionist and told she will be hearing from the clinic in 2 days to check on how she is doing.

### 5.3. How Should Suicidal Patients Be Followed-Up?

As with any acute medical condition, clinicians are responsible for assuring that care plans are executed in an appropriate and timely manner. Aside from arranging for subsequent appointments within their own clinic and reviewing whether the interventions they personally initiated are working, coordinating care with ancillary and specialist providers/facilities and communicating with the patient and their supporters are also vital (Box 8). If the patient is sent to the emergency room or for immediate outpatient mental healthcare and nothing is reported back, the clinician’s office should verify the patient arrived, was seen, and was admitted for inpatient care or discharged with subsequent psychiatric aftercare. If solely outpatient treatment is indicated, the clinician or their staff should contact the patient 24–48 h after the initial visit. Ask patients how they are feeling, review and adjust the safety plan as needed, scrutinize whether access to lethal means has been blocked [106], and check that they either have already been seen by a mental health professional or will be keeping the appointment made. Currently, up to 50% of suicidal patients do not show up for their psychiatric/psychological appointments [90]. These “caring contacts”—as fleeting and minimal as they might seem—have been shown to reduce suicidal ideation and behavior in some studies [90,107].

Box 8 **Clinical case—part 8.**

Two days later, Maria receives a telephone call from the medical assistant. The assistant asks her about how she is feeling and whether the psychiatrist or other clinicians have contacted her. Maria states that last night was the first night in 6 months she was able to obtain more than 4 h of continuous sleep. Her mouth feels a bit dry this morning, a mild side effect of the amitriptyline that the assistant tells her can be alleviated by chewing gum or drinking fluids. Maria forced herself to sit outside in the sun for 15 min and felt more cheerful afterwards. Dr Lopez’s office has scheduled her for a telephone call tomorrow. She has to return the social worker’s and occupational therapist’s voice messages. The assistant compliments Maria for spending time outside (part of her self-generated plan to address suicidal feelings) and for taking action.

The call is then transferred to the receptionist. Based on the severity of her ME/CFS, Maria is offered a virtual rather than in-person visit with the doctor in one week.

In Maria’s situation, the clinician should make a formal follow-up appointment (Box 9) with her for medication management (e.g., Was she able to obtain the amitriptyline? Has she tried them? What have the effects been?) and at the next visit, acknowledge any efforts she has put forth (e.g., “I know how hard it must be to reach out to strangers for help. You should be proud of yourself.”). Speaking briefly with or reviewing notes from the occupational therapist and social worker can update the clinician regarding the patient’s functional status, support network, and housing situation. Asking open-ended questions, letting the patient lead the conversation sometimes, and showing a personal interest in the patient can also support the patient on their path to better mental health. If a patient is discharged from inpatient psychiatric care, an outpatient appointment should be made within 7 days at most [108]. During the first week and first month after hospitalization, the risk of suicide is 200 and 300 times higher than the general population, respectively [109]. Even after 5 to 10 years, the rate of suicide was 30 times than of the general population. Thus, suicidality should be considered a chronic condition that the clinician inquires about occasionally even when the patient is stable, much as they would with hypertension or diabetes. Clinicians should also be aware of when patients stop or are discharged from outpatient psychiatric/psychological care.

Box 9 **Clinical case—part 9.**

The next week, Maria sees her doctor virtually, using online video-conferencing software. Her sleep continues to improve, especially as pain no longer wakes her up, and she has a little bit more energy to take care of herself and her household chores. She reports that during Dr Lopez’s phone visit, they discussed positive coping behaviors such as focusing on what she can do (vs. what she cannot do), setting small goals and working towards them. Her “homework” includes planning at least one social interaction (which is tolerable for her level of ME/CFS activity) every week and noting something she is grateful for every night in a journal. She will be talking to Dr Lopez again next week.

The doctor reviews the occupational therapist’s notes. Maria qualifies for bath and shower bars and a shower stool to decrease dizziness and exhaustion while bathing. A wheelchair is recommended to facilitate travel outside the home. The OT also teaches Maria to balance her activities with rest and to save her most challenging activities for the times of the day when she is likely to have more energy. This has allowed Maria to start completing an application for housing support mailed to her by the social worker.

Maria states she is starting to feel more optimistic about her future: if her symptoms continue to abate a little more and she can get around in a wheelchair, she might be able to attend a knitting group that her neighbor Sarah hosts twice a month.

The doctor celebrates her progress with her causing Maria to feel confident enough to ask the doctor whether orthostatic intolerance—which she has been able to read more about—might account for some of her nausea and dizziness. The doctor admits she does not know much about OI but will try to learn more about it. A follow-up appointment is scheduled in 2 weeks.

## 6. Barriers, Gaps, and Opportunities

We recognize that the process of assessment and treatment detailed in this article may be challenging to implement. Research, clinical, and societal barriers exist.

### 6.1. Research Barriers

Much more investigation into the relationships among suicide, chronic physical illness, and ME/CFS are needed. Oftentimes, concepts, assessments, and interventions had to be extrapolated from one field to another because studies were absent or lacking. For example, risk factors for suicidal ideation may not be the same as those for suicide attempts or completion. Motivation for suicide in ME/CFS or in chronic medical illnesses might be different from that of psychiatric patients or the general population. Epidemiological studies of suicide in ME/CFS have been retrospective in nature, either medical record reviews or psychological autopsies. That has meant the true incidence and prevalence of suicide are not known and drivers of suicide could be better elucidated. Most of what we know about ME/CFS itself is based on adult patients who are given an ME/CFS diagnosis and possess the health, financial and social status to access the few specialists scattered globally. Although the C-SSRS and the ASQ are helpful, their use by non-mental health professionals in adult patients seen in community-based, outpatient medical settings has not been well-established. Self-report assessments also have weaknesses: up to 25% of patients who attempted suicide actively denied suicidality during appointments the week before [110] due to shame, embarrassment and concerns about losing autonomy if they were hospitalized. Refinement of risk also relies on clinician interpretation of patients’ words. Consequently, some scientists are eagerly pursuing biomarkers which can predict risk [111]. There is adequate research in some areas of treatment, such as the efficacy of antidepressants or suicide-specific dialectical behavioral therapy, but less in others, such as the impact of occupational therapy on suicide risk when functional restrictions are present. Reviewing research for this paper reveals a plethora of questions and issues that have yet to be answered.

### 6.2. Clinical Care Barriers

Educational, attitudinal, logistical, financial, and legal barriers impede optimal care. Healthcare providers readily admit they are not confident about diagnosing and managing both ME/CFS [4] and suicidality [65,66]. Hopefully, the process outlined (Figure 1) and detailed in this article advances practitioners’ knowledge about suicidality and supplies them with a straightforward care plan. Widespread education of healthcare providers and the public about ME/CFS is a critical step in reducing stigma and the unsupportive social interactions driving suicidality in ME/CFS. The 2015 US National Academy of Medicine Report for Clinicians [4], 2014 International Association for Chronic Fatigue Syndrome/Myalgic Encephalomyelitis (IACFS/ME) Primer [33], US Centers for Disease Control and Prevention (CDC) ME/CFS website [112], and US ME/CFS Clinician Coalition short summary [113] provide guidance on the clinical care of ME/CFS.

As presented in this article, optimal outcomes necessitate multiple professionals working together with the patient. Coordination and communication among these individuals are vital yet often neglected. Multiple appointments for care separated by time and physical location are especially challenging for severely ill populations. Some organizations are examining whether mental health professionals embedded in medical practices, where they can see patients immediately, or specially trained personnel (e.g., a nurse or social worker) assigned to outpatient clinics can help [12,18]. Clinicians have also been concerned about whether protocols addressing suicide will take up too much time or resources. For both the ASQ and C-SSRS, in non-psychiatric settings, over 95% of screenings are rated as “no” or “minimal” risk (meaning no further action is needed), 1.9%, as moderate risk, and 0.2–0.5% as high risk [68,114]. The latter two percentages are likely higher among patients afflicted by ME/CFS yet may not be as overwhelming as expected. From an institutional point of view, standardized protocols have resulted in more cost-effective care as emergency rooms and mental health consultants channel their immediate energy towards patients at the highest risk rather than dispersing it among all patients at risk of suicide.

Health insurance reimbursement and coverage for mental health care continue to be obstacles. Despite mental health parity laws passed in the United States in 2008 and 2013 advocating for equal treatment of mental and physical health conditions [115], enforcement of regulations has not been consistent nor uniform across the country. For example, reimbursement for primary medical care is 30–50% higher than that for behavioral health care and prior authorizations obstruct timely access to care [116]. Circumstances are also challenging in Canada: the publicly funded health care system provides limited access to mental health professionals, such as psychologists and counselors [117]. Waiting lists and inability to access someone with illness-specific expertise are the norm. During 2020, the Canadian Alliance on Mental Illness and Mental Health introduced a new Mental Health Parity Act [118].

Some clinicians worry they will be held responsible for a patient’s completed suicide and thus avoid asking about suicide entirely. In the United States, the concepts governing liability in suicide are the same as to those affecting other medical conditions: existence of a duty, negligent breach of that duty, proven damage to the patient, and a proximate link between breach and damage [119]. Unfavorable outcomes by themselves do not necessarily lead to finding the clinician at fault. Instead, judgments are based on whether the professional acted reasonably according to community-accepted standards of care. The steps and questionnaires discussed in this article are based on the scientific literature and align with the 2018 guidelines from the US National Action Alliance for Suicide Prevention [90]. Constructing suicide safety plans may decrease legal exposure [120]. Careful, timely documentation of what was done and the rationale for decisions [15] as well as communicating the care plan to the patient’s family or supporters (with the patient’s written permission) can further protect against liability.

### 6.3. Societal Barriers

Ultimately, to decrease suffering and suicide in ME/CFS, steps must be taken on a larger, systemic level, beyond those of the research and clinical care realms. Education about ME/CFS must be extended to lawmakers and disability benefit providers, who have the authority to address the lack of resources so commonly experienced by those with ME/CFS and directly cited as a major factor in suicidal ideation [17]. Recently, some mental health professionals have pushed for programs responding to the external roots of suicide, including poverty, social connectedness, unemployment, firearm availability, and homelessness [121,122,123]. Expansion of such programs will also benefit patients with ME/CFS although some existing programs (e.g., Meals on Wheels) are unfamiliar with ME/CFS and thus, patients face skepticism when applying for them. Education of the public is also needed to reduce stigma and make the unsupportive social interactions driving suicidality in ME/CFS increasingly infrequent.

### 6.4. Emerging Opportunities

A potentially positive effect of the COVID-19 pandemic and lockdown is the expanded use of virtual care: teletherapy, telemedicine, etc. Home-based care, either in-person or virtual, is already being used in some homebound geriatric patients [124]; similar adaptations to those with ME/CFS who are home- or bed-bound could make accessible mental health services that might otherwise trigger too much post-exertional malaise to attend. In a randomized controlled trial of home-based care for people with multiple sclerosis, people who received care in the home showed significant improvements in multiple domains of quality of life, including the mental health-related role-emotional and social functioning domains [125]. Critically, this home-based care model integrated physical and mental healthcare, something important in ME/CFS given that physical symptoms such as sleep disturbance and pain are major risk factors for suicide, as previously discussed.

Even asynchronous online therapy has been shown in some studies to reduce suicidal ideation in primary care populations [126,127], and internet-based programs for comorbid depression and chronic illness show some success in reducing depression rates in meta-analysis [128]. Interventions delivered by telephone have also shown some success; an intervention for emergency department patients consisting of a safety plan, provision of crisis resources, and a series of telephone follow-ups reduced suicide attempts in the following year by 30% [129]. There are thus multiple feasible methods of mental healthcare delivery other than traditional in-person office visits, which could improve access to such care in the future. Adapting mental and physical healthcare to the energy limitations of people with ME/CFS represents a logical next step in treating this illness and is not unprecedented elsewhere in suicide prevention and chronic illness literature.

## 7. Conclusions

Like other chronic, debilitating illnesses, ME/CFS places individuals at an increased risk of death by suicide. Several characteristics prominent in ME/CFS exacerbate this risk and make diagnosis and management of suicidality demanding. These include absence of any disease-modifying treatments, severe functional limitations confining sizable numbers of patients at home, and symptoms (e.g., PEM, medication sensitivities, cognitive dysfunction) limiting certain therapies. Decades-long misattribution of ME/CFS to physical deconditioning or irrational, hypochondriacal beliefs combined with conflation of ME/CFS with depression or anxiety have also resulted in an uneducated healthcare workforce at best and a skeptical, dismissive one at worst. Severity of impairment is often not acknowledged. Consequently, some patients are reluctant to engage in psychiatric/psychological care despite sometimes desperately needing it. Lack of proper recognition by medical professionals and authorities in turn has meant an absence and scarcity of resources targeted or available to patients, whether medical/psychiatric/psychological care, social support from family members or friends, or disability benefits.

Outpatient medical professionals play a vital role in ameliorating this cascade of effects. We have provided a framework for identifying and managing adult suicidal patients afflicted by ME/CFS through adapting current recommendations to this neglected population. Through both applying evidence-based interventions aimed at all suicidal patients and tailoring interventions specific to an individual patient’s circumstances (Box 10), we believe that suffering and suicidality can be alleviated.

Box 10 **Clinical case—part 10.**

Three months later, Maria returns for a follow-up visit. Although her neck and arm pain persist, a higher dose of amitriptyline has dulled it considerably and she is able to sleep through the night now. Dr Lopez has her taking a stable dose of citalopram; she continues to see him. After being diagnosed with OI by her doctor, her doctor teaches her to mix up a homemade oral rehydration solution. Drinking this regularly helps control her dizziness and she is now able to sit up for 2 h at a time. With the help of the wheelchair, she is able now to attend Sarah’s knitting group regularly.

After her housing voucher application is approved, she is able to move to a new place, decreasing her respiratory symptoms. The extra financial assistance also allows her to save money each month.

Although Maria’s ME/CFS remains, symptom relief, treatment of depression, mild functional improvement, social connection, and a change in housing result in a decrease in suicidal ideation. Eventually, Maria joins a Facebook support group for patients with ME/CFS. As time passes, she is able to offer support and hope to new members. This gives her a renewed sense of purpose.

## Figures and Tables

**Table 1:** Risk factors for suicide.

Potentially Modifiable	Non-Modifiable
Chronic, serious illness ^1^ Sleep disturbances/problems Pain Other severe symptoms (e.g., cognitive dysfunction, hypersensitivity to stimuli) Depression Anxiety Substance Abuse Other comorbid medical conditions (e.g., fibromyalgia, orthostatic intolerance syndromes) Low quality of life Limited function ^1^ Social isolation, loneliness ^1^ Lack of supportive relationships ^1^ Thwarted belongingness ^1^ Unstable, challenging social circumstances (e.g., homelessness, poverty, unemployment) ^1^ Unsupportive social and healthcare provider interactions ^1^ Lack of/poor coping skills Personal beliefs	Older age Male sex Caucasian Native American/Alaskan Native background Identifying as LGBTQ ^2^ History of self-harm History of suicide attempts Recently discharged from inpatient psychiatric care Personality disorder Past traumatic events (e.g., adverse childhood experiences, sexual abuse, domestic violence) Family history of suicide, mental health disorder Exposure to other people who have committed suicide

^1^ Risk factors specifically cited by patients with myalgic encephalomyelitis/chronic fatigue syndrome; ^2^ Lesbian, gay, bisexual, trans, queer/questioning.

**Table 2:** Concerning statements, symptoms, behaviors, and events should prompt clinicians to assess for suicidality.

**Statements**
Passive suicidal ideation: “I wish I could go to sleep one day and not wake up.”
Active suicide ideation: “I am tired of living and looking for a way out.”
Depression: “I feel sad/cry all the time.”
Feeling like a burden to family/others: “My family would be better off if I were dead.”
Hopelessness: “I have nothing to look forward to.” “Life is meaningless.”
Loneliness: “There is no one I can talk to about my problems.” “I don’t have any friends.”
**Symptoms**
Changes in mood, including onset/exacerbation of depression anxiety; dramatic fluctuations
Worsening somatic symptoms, especially pain and insomnia
Anger, irritability
**Behaviors**
Agitated actions: pacing, shaking, rapid/loud speech
Impulsive behaviors
Withdrawal from care: stopping treatments, missing appointments, avoiding contact
Repetitive self-harm
Drinking or abusing other substances more than usual
Decreasing social contact
Giving away items which are important/meaningful to patient
Ceasing activities previously enjoyed
**Events**
Unemployment
Loss of significant relationships (e.g., divorce, death of loved one)
Denial of disability benefits
Homelessness
Anticipated treatment is not effective
Recent suicide attempt
Recent discharge from inpatient/outpatient psychiatric care

**Table 3:** Protective factors for suicide.

Potentially Modifiable	Non-Modifiable
Religious background/personal beliefs	Younger age
Positive coping behaviors	Female Sex
Strong relationships	Having children
Stable social circumstances (e.g., financial status, housing)	Marriage
Supportive clinical interactions	Pregnancy

**Table 4:** Suicide safety plan by Brown and Stanley.

Component	Ask Patient	Example Answers	Comment
1. Warning signs	How will you know when the safety plan should be used?	“Feeling hopeless.” “Thinking life is all downhill from here.” “Lying in bed more than usual.”	Thoughts, behaviors, moods, events that lead to suicidality.
2. Internal strategies	What activities can you do on your own if you become suicidal again, to help yourself not to act on your thoughts or urges?	Sit outside in the sun, listen to relaxing music, take a warm bath.	
3. People and settings that provide distraction	Who helps you take your mind off your problems at least for a little while? Where can you go where you will be around people in a safe environment?	Knitting group, the park near my home, online patient support group.	People named need not know about the patient’s suicidal feelings. Places may allow casual interactions.
4. People whom I can contact for help	Who is supportive of you and who do you feel that you can talk with when you are under stress?	My neighbor Sarah, my church’s pastor.	These are people who are aware of or could be trusted with the individual’s suicidal thoughts/feelings.
5. Professionals and agencies I can call in a crisis	Who are the medical/mental health professionals that we should identify to be on your safety plan?	Springfield Emergency Room, my psychiatrist Dr Joseph Lopez, National Suicide Prevention Lifeline, 911	List contact information.
6. Making the environment safe	What items do you have around you that you might use to hurt/kill yourself? How can we make your surroundings safe for you?	Doctor/pharmacy will limit number of medications mailed to one week at a time. Place kitchen knives in locked cabinet.	Always ask about firearms. Means restriction should be matched to the methods the individual names.
7. My reasons for living ^1^	What makes your life worth living? What brings joy to your life?	My children, my faith, my pets, enjoying nature.	

^1^ Except for this step, all others are drawn from Brown and Stanley’s work on suicide safety planning. Adapted with permission from Dr Barbara Stanley, Cognitive and Behavioral Practice, published by Elsevier, 2012 [92]. Please see Figure S1 in Supplementary Materials or suicidesafetyplan.com (accessed on 17 May 2021) for a downloadable template which can be used with patients.

**Table 5:** Interventions addressing individual-specific risk factors for suicide.

Category	Examples of Specific Factor	Examples of Interventions	Comments
ME/CFS ^1^ symptoms	Sleep Pain	Cognitive behavioral therapy—insomnia Blue light filters Exposure to natural light ^2^ Amitriptyline ^3^ Trazodone ^3^ Re-positioning Massage Heat/ice Gabapentin ^3^ Tricyclic antidepressant ^3^	Evaluate for pain and sleep conditions with specific treatments (e.g., obstructive sleep apnea, migraine).
Comorbid psychiatric conditions	Major depressive disorder	Referral to mental health professional CBT ^4^ Citalopram ^3^ Venlafaxine ^3^	
Comorbid medical conditions	Multiple chemical sensitivity Postural orthostatic tachycardia syndrome (POTS)	Avoid/reduce exposure to concerning stimuli Isotonic fluids, support hose, awareness/prevention of exacerbating factors, recumbent exercises, fluoxetine ^3^	Exercise may not be suitable for many patients. If used, start at a low level and continue/increase only if patient tolerates.
Isolation/loneliness/social support	Healthcare professionals Family/caregiver Community support	Validation of patient experience Reflective listening Caring contacts Educate about ME/CFS Educate about caregiver stress In-person activity/support groups Electronic forums specific for ME/CFS Virtual support groups	Caring contacts are brief, intermittent e-mails, cards, phone calls to patients by staff between visits. Caregivers need respite/support to provide support.
Functional Limitations	Ambulation Bathing	Refer to physical therapy Bedside commode Wheelchair Refer to occupational therapy Hand-held shower head Shower chair	
Other Support	Poverty Homelessness	Food banks, vouchers Apply for disability financial support Home-sharing/roommate arrangements Government-supported housing vouchers	Clinic/facility-based medical social workers can help patients find and apply for programs.

^1^ Myalgic encephalomyelitis/chronic fatigue syndrome. ^2^ For some patients, especially the severely ill, bright light may worsen their ME/CFS. For others, light sensitivity is not a problem or is tolerable with sunglasses. ^3^ Start all medications at lower dosages and titrate up slowly. Pain, sleep, and sedative medications may need to be given in smaller quantities (e.g., a week’s supply) initially due to risk of suicide. ^4^ Cognitive behavioral therapy.

## Supplementary Materials

The following are available online at https://www.mdpi.com/article/10.3390/healthcare9060629/s1, Figure S1: Suicide Safety Plan Template. Reproduced with permission from Dr Barbara Stanley, Cognitive and Behavioral Practice, published by Elsevier, 2012 [92].
