# Supplementary material for: Identifying and Managing Suicidality in Myalgic Encephalomyelitis/Chronic Fatigue Syndrome
Source: Healthcare (Basel). 2021 May 25;9(6):629. doi: 10.3390/healthcare9060629 (PMC8227525; doi:10.3390/healthcare9060629)
Supplement: Supplementary file 1 [file healthcare-09-00629-s001.zip › healthcare-1184427-supplementary.pdf]

SAFETY PLAN

Step 1: Warning signs:

1. ____
2. ____
3. ____

Step 2: Internal coping strategies - Things I can do to take my mind off my problems without contacting another person:

1. ____
2. ____
3. ____

Step 3: People and social settings that provide distraction:

1. Name________________________ Phone____________
2. Name________________________ Phone____________
3. Place____
4. Place ____

Step 4: People whom I can ask for help:

1. Name________________________ Phone____________
2. Name________________________ Phone____________
3. Name________________________ Phone____________

Step 5:Professionals or agencies I can contact during a crisis:

1. Clinician Name____________________ Phone______________

   Clinician Pager or Emergency Contact #______________________
2. Clinician Name____________________ Phone______________

   Clinician Pager or Emergency Contact #______________________
3. Suicide Prevention Lifeline: 1-800-273-TALK (8255)
4. Local Emergency Service ______________________________

   Emergency Services Address____________________________

   Emergency Services Phone _____________________________

Making the environment safe:

1. ____
2. ____

From Stanley, B. & Brown, G.K. (2011). Safety planning intervention: A brief intervention to mitigate suicide risk. *Cognitive and Behavioral Practice. 19*, 256–264
